# Fluoride-Releasing Self-Etch Adhesives Create Thick ABRZ at the Interface

**DOI:** 10.1155/2021/9731280

**Published:** 2021-07-29

**Authors:** Toru Nikaido, Tomohiro Takagaki, Takaaki Sato, Michael F. Burrow, Junji Tagami

**Affiliations:** ^1^Department of Operative Dentistry, Division of Oral Functional Science and Rehabilitation, School of Dentistry, Asahi University, 1851, Hozumi, Mizuho City, Gifu 501-0296, Japan; ^2^Department of Cariology and Operative Dentistry, Graduate School of Medical and Dental Sciences, Tokyo Medical and Dental University (TMDU), 1-5-45 Yushima, Bunkyo-Ku, Tokyo 113-8549, Japan; ^3^Restorative Dental Sciences, Faculty of Dentistry, University of Hong Kong, Hong Kong, Pokfulam, Hong Kong SAR, China

## Abstract

A fluoride-releasing adhesive system is expected to promote mineralization of demineralized dentin/enamel around a composite restoration, thereby contributing to the longevity of the restoration. Scanning electron microscopic (SEM) and transmission electron microscopic (TEM) observations revealed the formation of an “acid–base resistant zone” (ABRZ) beneath the hybrid layer when dentin was treated with a self-etch adhesive system. A thicker ABRZ was formed from the upper slope to the end of the outer lesion, indicating greater resistance against an acid-base challenge, when a fluoride-releasing self-etch adhesive system was used. The slope formation of a fluoride-releasing adhesive is believed to be due to fluoride-release from the adhesive. Quantitative assessment of the acid resistance was performed at the interface using the region of interest (ROI) mode of a digital image analysis software package. The area of the ABRZ is influenced by the concentration of fluoride release from the adhesive resin. The threshold of fluoride concentration in the adhesive may exist to influence the morphology of the ABRZ. X-ray absorption fine structure (XAFS) analysis of the dentin treated with different concentrations of NaF-mouth rinses suggested that different fluoride concentrations result in the formation of different chemical compounds, such as fluorapatite and CaF_2_-like structures, on the dentin surface. This may explain the differences in *μ*TBS values and morphological appearance of the ABRZ. NaF is effective in enhancing the enamel/dentin bond durability and also helps create a high quality of ARBZ to improve the clinical success of restorations.

## 1. Introduction

Fluoride has been shown to exhibit anticariogenic activity by increasing enamel and dentin resistance to acid attack as well as inhibit carbohydrate metabolism in dental plaque [1, 2]. Also, fluoride is a vital component in the remineralization process which reduces the rate of demineralization of dental tissues should the biofilm become cariogenic [3–5].

A fluoride-releasing adhesive system is expected to promote the mineralization of demineralized dentin/enamel around a composite restoration, thereby contributing to the longevity of the restoration [6–8]. In contrast, the application of fluoride to tooth substrates prior to bonding is a controversial issue, because increasing acid resistance of tooth structure may reduce the etching effect, particularly with self-etch adhesive systems [9].

Ultrastructural examinations of the adhesive–dentin interface after an acid–base challenge have been carried out to investigate the mechanism of recurrent caries at the margins of restorations [10, 11]. This literature review describes the effect of fluoride-containing adhesives on resin-tooth interfacial morphology.

## 2. Ultrastructural Examinations of the Adhesive–Dentin Interface

**Figure 1:**
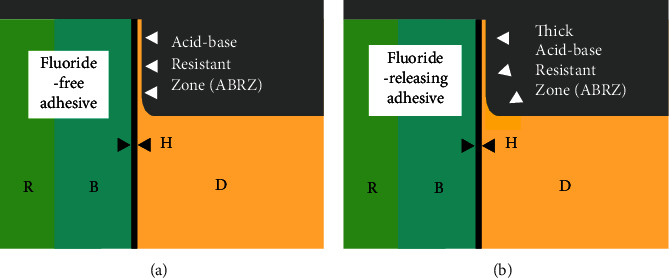
Illustrations explaining the formation of acid-base resistant zone (ABRZ) with fluoride-free self-etch adhesive (a) and fluoride-releasing adhesive (b). Self-etch adhesives create an ABRZ beneath the hybrid layer (H); however, the fluoride-releasing adhesive creates a thicker ABRZ than the fluoride-free adhesive. B: bonding resin; D: dentin; R: resin composite.

Scanning electron microscopic (SEM) and transmission electron microscopic (TEM) observations have revealed the formation of an “acid–base resistant zone” (ABRZ) found beneath the hybrid layer when dentin was treated with a self-etch adhesive system [12, 13]. The morphology of the ABRZ has been shown to be influenced by the composition of the adhesive system [14–17].  Figure 1 illustrates the formation of the acid-base resistant zone (ABRZ) with a fluoride-free self-etch adhesive (a) and a fluoride-releasing adhesive (b). Self-etch adhesives create an ABRZ beneath the hybrid layer (H). The thickness of the ABRZ is different between a fluoride-free adhesive and a fluoride-releasing adhesive. Generally, when a fluoride-releasing self-etch adhesive system is used, a thicker ABRZ is formed when observed from the upper slope to the end of the outer lesion, indicating greater resistance against an acid-base challenge (Figure 1(b)) [18, 19].

## 3. Fluoride-Releasing 2-Step Self-Etch Adhesive Systems

**Figure 2:**
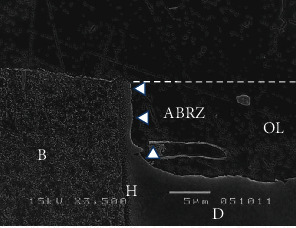
SEM observation of the interface between Clearfil Protect Bond and dentin after acid-base challenge (× 3,500). The ABRZ was sloped from the top to bottom, approaching the dentin. B: bonding resin; D: dentin; H: hybrid layer; OL: outer lesion; R: resin composite.

**Figure 3:**
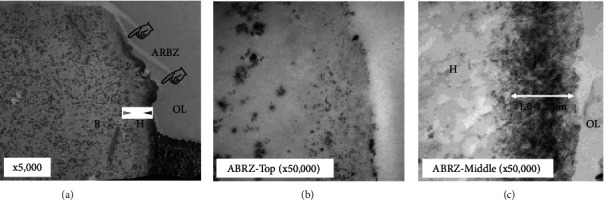
TEM observations of the interface between Clearfil Protect Bond and dentin after acid-base challenge ((a) ×5,000, (b) ×50,000, (c) ×50,000). B: bonding resin; D: dentin; H: hybrid layer; OL: outer lesion. An ABRZ (finger pointers) was observed beneath the hybrid layer (H) at lower magnification (a). Apatite crystals were sparce at the top of the ABRZ (b), while apatite crystals at the middle of the ABRZ were much greater in quantity (b). The thickness of the ABRZ was approximately 1.0-1.2 *μ*m.

The two-step self-etch adhesive system, Clearfil Protect Bond (PB, Kuraray-Noritake Dental, Tokyo, Japan) is composed of a self-etching primer and a fluoride-containing adhesive [18]. The bonding agent of PB contains specially treated sodium fluoride crystals which can release fluoride ions from the adhesive [20].  Figure 2 shows an SEM image of the interface between PB and dentin after acid-base challenge [20]. The ABRZ is sloped from the top to bottom as it approaches the dentin.  Figure 3 shows TEM images of the interface between PB and dentin after the acid-base challenge [21]. An ABRZ (finger pointers) was observed beneath the hybrid layer (H) at lower magnification (Figure 3(a)). The thickness of the hybrid layer (H) was less than 1 *μ*m. Apatite crystals were sparse at the top of the ABRZ (Figure 3(b)) and were noted to be much greater in quantity at the middle of the ABRZ (Figure 3(c)). The thickness of the ABRZ was approximately 1.0-1.2 *μ*m.

**Figure 4:**
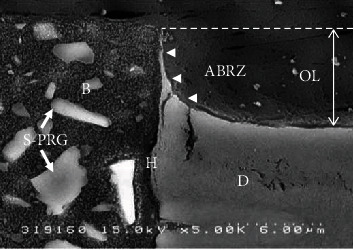
SEM observation of the interface between Fluorobond II and dentin after acid-base challenge (× 3,500). The different sizes of the S-PRG filler particles (white arrows) were observed in the adhesive resin (B). The ABRZ was sloped from the top to the bottom, as it approached the underlying dentin. B: bonding resin; D: dentin; H: hybrid layer; OL: outer lesion.

A two-step self-etching primer system, FL-Bond II (FL II, Shofu, Kyoto, Japan) contains a multi-ion releasing component made up of surface pre-reacted glass ionomer (S-PRG) fillers. The S-PRG filler-containing adhesive of FL II is able to release various ions, such as aluminum (Al^3+^), boron (BO_3_^3-^), fluoride (F-), sodium (Na+), silicon (SiO_3_^2-^), and strontium (Sr^2+^), in both neutral and acidic conditions [22–24].  Figure 4 shows an SEM image of the interface between FL II and dentin after acid-base challenge [25]. Different sizes of the S-PRG filler particles (white arrows) were observed in the adhesive resin. The ABRZ of FL-Bond II was also sloped and exhibited an increased in thickness from the surface to the base of the outer lesion. This particular slope formation observed for FL II is believed to be due to fluoride release from the adhesive [26].

## 4. Thickness of the ABRZ with Different Concentration of NaF in the Adhesive Resin

**Figure 5:**
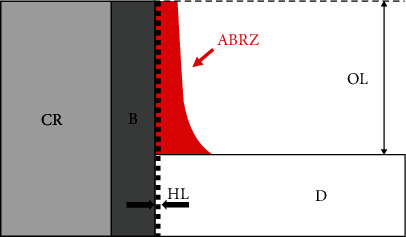
Schematic illustration of the area of the ABRZ. OL: outer legion; D: dentin; B: bonding resin; CR: composite resin; HL: hybrid layer. The red part is the ABRZ. The area was measured with ROI mode after SEM observation.

The concentration of NaF in the adhesive resin influenced the amount of dentin structure remaining after acid-challenge in the two-step self-etching adhesive system. Kirihara et al. [20] performed a quantitative assessment of the acid resistance at the interface using the region of interest (ROI) mode of digital image analysis software (Figure 5). Seven experimental adhesives with different concentrations of NaF (0 wt%; F0 to 100 wt%: F100) were prepared based on the formulation of a commercially available adhesive (Clearfil Protect Bond, F100). There was a significant difference in the ABRZ area observed between F20 and F50 and between F75 and F90, respectively. The fluoride concentration had to reach a threshold in order to form the slope. They suggested that the threshold of fluoride concentration in the adhesive to influence the morphology of the ABRZ should ideally be between F20 and F50.

## 5. Additive Effect of NaF on Mechanical Properties of Adhesive Resin

Nakajima et al. [27] reported that PB demonstrated stable dentin bond strengths after 1 year of storage in water, while Clearfil SE Bond (SE, Kuraray-Noritake Dental) without fluoride release demonstrated a decrease in the bond strength over the same period of time. Both SE and PB contain the acidic functional monomer, 10-methacryloxydecyl dihydrogen phosphate (MDP) [28, 29]. Matsui et al. [30] evaluated the role of MDP in the bonding resin of a 2-step self-etch system on dentin bonding performance. They reported that the addition of MDP in the adhesive resin improved the immediate dentin bond strength; however, MDP presence was associated with a reduction in long-term dentin bond durability. It is suggested that bond degradation may be accelerated by the presence of MDP in the bond.

Kakiuchi et al. [31] reported that the addition of NaF may be a key factor to prevent reduction of the enamel bonding performance. The addition of MDP in the adhesive may result in rapid water sorption because of the hydrophilic group of the phosphoric acid ester group of MDP. The addition of NaF into the adhesive may prevent water sorption by the adhesive. They evaluated the effect of MDP and NaF in the adhesive resin on the bonding performance of the two-step self-etch adhesive to enamel. They demonstrated that the bond strength gradually decreased with the MDP-containing adhesive systems; however, with the addition of NaF into the MDP-containing adhesive system, it was demonstrated that the enamel bonding performance became much more stable. They reported that the MDP-containing adhesive created an enamel ABRZ with erosion at the interface [32]. However, the NaF-containing adhesive was able to create a thick enamel ABRZ without erosion at the interface. They concluded that NaF is very effective in enhancing enamel bond durability and also significantly improving the quality of the enamel ARBZ.

## 6. Fluoride Application prior to Bonding with Fluoride-Free Adhesive

High concentrations of NaF are believed to be effective in increasing the acid resistance of the dentin [33, 34] and possibly in reducing the effect of the self-etching primer. Therefore, the application of fluoride to tooth substrates prior to bonding is a controversial issue, since increasing the acid resistance of tooth structures may prevent or severely reduce the etching of a self-etch adhesive system.

Nakamoto et al. [35, 36] evaluated the effect of fluoride mouth rinses on dentin bond strength using a fluoride-free self-etch adhesive system. The results indicated that the 450 ppmF and 900 ppmF NaF-containing mouth rinses did not affect bonding to dentin, while a 9000 ppmF NaF mouth rinse decreased dentin bonding performance. A change in the degree of slope formation of ABRZ was created by the application of different concentrations of NaF mouth-rinses to the dentin surface before bonding with a fluoride-free adhesive.

X-ray absorption fine structure (XAFS) analysis [23] of dentin treated with NaF-mouth rinse suggested the formation of fluorapatite-like structures on the dentin surface treated with the 450 and 900 ppm F fluoride mouth rinse solutions; in contrast, different XAFS spectra, which appeared to be similar to that of CaF2, were detected in the specimens treated with the 9000 ppm F solution. These findings implied that different fluoride concentrations result in the formation of different chemical compounds on the dentin surface and may explain the differences in *μ*TBS values and morphological appearance of the ABRZ in this study. In addition, this study suggested that fluoride incorporation into an adhesive could influence the slope formation of ABRZ. The theory for the reduced tendency of the apatite crystal dissolution in the presence of fluoride ions may be applicable for the formation of the thick ABRZ observed with the fluoride-releasing adhesive systems. The formation of acid-resistant fluoroapatite may be another possibility.

Abbassy et al. [37] reported that a fluoride-containing bioactive glass paste as a temporary filling material improved enamel/dentin bond durability and also remineralized the demineralized tooth structures, which may be another possible approach to apply fluoride to tooth structures prior to bonding of a fluoride-free adhesive system.

## 7. Conclusions

A fluoride-releasing adhesive has the potential to create a thick ABRZ at the adhesive interface, protecting restoration margins from recurrent caries initiation. However, the area of the ABRZ is influenced by the concentration of fluoride ion release from the adhesive resin. NaF is effective in enhancing the enamel/dentin bond durability and also creating a high quality of ARBZ to improve the clinical success of restorations.
